# Effects of Maternal and Progeny Dietary Vitamin E on Growth Performance and Antioxidant Status of Progeny Chicks before and after Egg Storage

**DOI:** 10.3390/ani11040998

**Published:** 2021-04-02

**Authors:** Jun Yang, Keying Zhang, Shiping Bai, Qiufeng Zeng, Jianping Wang, Huanwei Peng, Yue Xuan, Zhuowei Su, Xuemei Ding

**Affiliations:** Institute of Animal Nutrition, Key Laboratory for Animal Disease-Resistance Nutrition of China Ministry of Education, Sichuan Agricultural University, 211 Huimin Road, Wenjiang District, Chengdu 611130, Sichuan, China; yangjun0612@163.com (J.Y.); shipingbai@sicau.edu.cn (S.B.); zqf@sicau.edu.cn (Q.Z.); wangjianping1983@hotmail.com (J.W.); phw@sicau.edu.cn (H.P.); xuanyuede1007@hotmail.com (Y.X.); wzs698@126.com (Z.S.)

**Keywords:** maternal vitamin E, egg storage, offspring, performance, antioxidant status

## Abstract

**Simple Summary:**

Prolonged egg storage duration has been indicated to decrease the quality of hatchlings and the growth performance of offspring. Maternal nutrition plays a vital role in growth of chicks post-hatch. However, no work has been performed to evaluate whether or not maternal nutrition could improve the growth performance of offspring hatched from stored eggs. Here, we aimed to investigate the effects of maternal and progeny dietary vitamin E supplementation on the growth performance and antioxidant status of offspring before and after egg storage. Our results showed that maternal dietary vitamin E (VE) supplementation of 200 or 400 mg/kg could improve the growth performance and antioxidant status of offspring hatched from stored eggs, but not for that of offspring hatched from unstored eggs. These findings suggested that maternal dietary vitamin E was beneficial to improve the quality of long-term storage eggs.

**Abstract:**

Two trials were conducted to investigate the effects of maternal and progeny dietary vitamin E (VE) supplementation on the growth performance and antioxidant status of offspring before and after egg storage. A total of 576 75-week-old Ross 308 breeder hens were assigned to three dietary VE treatments (100, 200, and 400 mg/kg) with 6 replicates of 32 hens for 12 weeks. Two trials were conducted with offspring hatched from eggs laid at weeks 9 and 12 of breeder feeding trial, respectively. Trial 1 was conducted by a 3 × 2 factorial arrangement of treatments with three levels of maternal dietary VE (100, 200, and 400 mg/kg) and two levels of progeny dietary VE (0 and 35 mg/kg). Trial 2 was conducted with three maternal dietary VE treatment (100, 200, and 400 mg/kg), and chicks were hatched from eggs stored for 14 d and received the same progeny diet with no addition of VE. Results showed that in trial 1, maternal (100, 200, and 400 mg/kg) and progeny (0 and 35 mg/kg) dietary VE supplementation did not affect the growth performance of offspring hatched from unstored eggs (*p >* 0.05). In trial 2, in the case of long-term egg storage, maternal dietary VE supplementation of 200 and 400 mg/kg increased the body weight (BW) of 21- and 42-d-old offspring and the body weight gain (BWG) of offspring from 1 to 21 d (*p <* 0.05), and decreased the feed conversion ratio (FCR) of offspring from 1 to 21 d (*p <* 0.05) compared to 100 mg/kg VE. As the maternal dietary VE levels increased, the liver and serum antioxidant status of offspring enhanced (*p <* 0.05). In conclusion, maternal dietary VE supplementation of 200 or 400 mg/kg could improve the growth performance and anti-oxidant status of offspring hatched from stored eggs, but not for that of offspring hatched from unstored eggs. The suitable VE level for the broiler breeder diet was 400 mg/kg in the case of long-term egg storage.

## 1. Introduction

The antioxidant system of poultry consists of enzymatic antioxidant systems, such as superoxide dismutase (SOD), catalase (CAT), and glutathione peroxidase (GSH-Px), and non-enzymatic antioxidant systems, such as vitamin A, vitamin E, vitamin C, carotenoids, glutathione, selenium, and coenzyme Q [1]. As an important lipid-soluble antioxidant, vitamin E (VE) plays a positive role in protecting tissues and organs from oxidative damage by free radicals [2,3]. Recent studies on the broiler breeders demonstrated that dietary VE supplementation (0~150 mg/kg) did not affect the performance of breeders, and the fertility and hatchability of eggs [4,5,6]. Hossain et al. [7] found that the egg production of breeder, hatchability of eggs, and the growth performance of offspring were not influenced by the broiler breeder dietary VE supplementation (25, 50, 75, and 100 mg/kg). In broilers, dietary supplementation of 100~200 mg/kg VE did not affect the growth performance of broilers compared with no addition of VE [8,9]. It is not clear whether there is an interaction between maternal and progeny dietary VE levels on the growth performance of offspring.

Storing fertilized eggs is a frequent practice in the poultry industry. Prolonged egg storage (beyond 7 days) could induce the apoptosis of blastodermal cells [10,11,12] and delay the embryonic development [13,14], and result in an increase in the number of dead embryos, especially at the early and late stages of incubation [15,16]. As a result, the hatchability of eggs, quality of newly hatched chicks, and their growth performance post-hatch decreased [17,18,19,20,21,22]. The strategies of preincubation prior to storage [20,23], turning eggs during storage [21,24], short periods of incubation during storage [15,23], and altering egg position during storage [25], have had varying levels of success to improve the hatchability and chick quality following long-term egg storage. Dymond et al. [15] further observed that the short periods of incubation during egg storage improved the growth performance of offspring. Our previous studies have demonstrated that maternal dietary VE supplementation increased the hatchability and the antioxidant status of embryos and newly hatched chicks in the case of long-term egg storage [26,27]. Yet, no work has been performed to evaluate whether or not increased maternal dietary VE supplementation could further improve the growth performance of offspring hatched from stored eggs, as eggs were commonly stored in commercial poultry production. Thus, the aim of the present study was to investigate the effects of maternal and progeny dietary VE supplementation on the growth performance and antioxidant status of offspring before and after egg storage.

## 2. Materials and Methods

All animal procedures used in this study were approved by the Animal Care and Use committee, Sichuan Agricultural University (Ethic Approval Code: SICAUAC201710-7; Chengdu, China).

### 2.1. Experimental Design and Diets

The feeding trial of broiler breeder was conducted at Yuguan breeding farm, located at Shehong city (Sichuan Province, China). A total of 576 75-week-old Ross 308 breeder hens were assigned to three dietary treatments with 6 replicates of 32 hens. Breeder hens were fed a basal mash diet (Table 1) supplemented with 100, 200, or 400 mg/kg VE (the dose of 100 mg/kg was the nutrient recommendation of Ross 308 parent stock) for 12 weeks. The management of breeders was the same as the description of Yang et al. [26].

Two trials were conducted with progeny chicks hatched from eggs laid at weeks 9 and 12 of the breeder feeding trial. In trial 1, qualified eggs were collected for 5 consecutive days and incubated using a commercial incubator (Pearl-22, I.P Co., Ltd., Tokyo, Japan). The temperature of the dry-bulb was initially set at 37.9 °C and gradually decreased down to 36.5 °C as the time of incubation time increased; the wet-bulb temperature was maintained at 28.2 °C. After 19 days incubation, the eggs were diverted into a hatcher with the dry-bulb set at 36.9 °C and the wet-bulb set at 29.0 °C until incubation. At hatch, 240 healthy chicks from each maternal dietary VE treatment were randomly selected and divided into 12 pens with 20 chicks per pen. The 12 pens per maternal treatment were randomly assigned to 1 of 2 progeny dietary VE (0 and 35 mg/kg; the dose of 35 mg/kg was the common value in commercial broiler production of China) treatments for 42 days. In trial 2, qualified eggs were collected for 5 consecutive days and then stored for an additional 14 days in a room with controlled temperature of 16–18 °C and 75% RH until incubated. At hatch, 130 healthy chicks from each maternal dietary VE treatment were randomly selected and divided into 5 pens with 26 chicks per pen. All of the chicks were fed the same diet with no addition of VE for 42 days.

Chicks in the two trials received the same commercial basal diets (Table 1). The house temperature was initially set at 33 ± 1 °C and then gradually reduced to 21 ± 22 °C after 35 days with a reduction of 0.3 °C per day. Water and feed were supplied ad libitum. Two trials were conducted in a commercial broiler farm located at Shehong city (Sichuan Province, China).

### 2.2. Body Weight and Feed Consumption

Body weight (BW) and feed intake (FI) for each pen were measured on day 22 and 43 in trial 1 and 2. The body weight gain (BWG) and adjusted feed conversion ratio (FCR) based on mortality per pen were calculated.

### 2.3. Sample Collection and Analysis

On day 22 and 43 in both trials, one bird per replicate was randomly selected for blood sampling via the jugular vein. Blood samples were centrifuged at 1200× *g* for 10 min at 4 °C to obtain the serums, which were immediately stored at −20 °C until further analysis. Then, these birds were euthanized by cervical dislocation for liver (stored at −20 °C) sample collection. Liver samples were mixed at a ratio of 1:9 with physiological saline to make tissue homogenates. Homogenates were then centrifuged at 1500× *g* for 10 min at 4 °C to obtain the supernatant fluid. The determination of serum and liver malondialdehyde (MDA), total superoxide dismutase (T-SOD), and total antioxidant capacity (T-AOC) was according to the description of our previous study [26] and the methods of specific as-say kits, purchased from the Nanjing Jiancheng Bioengineering institute of China.

### 2.4. Statistical Analyses

Data in trial 1 were analyzed by ANOVA as a 3 × 2 factorial using GLM procedures of SPSS 21.0 (SPSS Inc., Chicago, IL, USA). The main effects (maternal and progeny dietary VE supplementation) and interactions between the two factors were carried out. Duncan’s test was applied when any of the interactions showed significance. Data of growth performance in trial 2 were analyzed by one-way ANOVA using SPSS 21.0 (SPSS Inc., Chicago, IL, USA). When the ANOVA showed significance, Duncan’s significant-difference test was applied. Data of antioxidant status in trial 2 were analyzed by ANOVA as a 2 × 2 factorial using GLM procedures of SPSS 21.0 (SPSS Inc., Chicago, IL, USA). The main effects (progeny age and maternal dietary VE supplementation) and interactions between the two factors were carried out. Duncan’s test was applied when any of the interactions showed significance. Linear and quadratic effects of maternal dietary VE levels in trial 2 were analyzed. Data were shown as the means and pooled standard error of the mean (SEM). The results were considered significantly different at *p* < 0.05.

## 3. Results

### 3.1. Growth Performance and Antioxidant Status in Trial 1

Results presented in Table 2 indicated that maternal and progeny dietary VE showed no significant influence on the growth performance of offspring from 1 to 42 d of age (*p >* 0.05).

An interaction was found between maternal and progeny dietary VE on the liver T-AOC of 42-d-old offspring (*p <* 0.05; Table 3). No difference between maternal VE treatments was observed on the liver T-AOC of 42-d-old offspring with no addition of VE in progeny diet (*p >* 0.05). However, the liver T-AOC of 42-d-old offspring increased as maternal dietary VE supplementation increased with addition of 35 mg/kg VE in progeny diet (*p <* 0.05).

### 3.2. Growth Performance and Antioxidant Status in Trial 2

As shown in Table 4, maternal dietary VE levels linearly and quadratically increased the BW of 21-d-old offspring (*p <* 0.05) and the BWG of offspring from 1 to 21 d (*p <* 0.05); and linearly and quadratically decreased the FCR of offspring from 1 to 21 d (*p <* 0.05). There were linear and quadratic increases in BW of 42-d-old offspring and BWG of offspring from 1 to 42 d to increasing maternal dietary VE levels (*p <* 0.05). Although maternal dietary supplementation of 200 and 400 mg/kg VE increased the BW of 28-d-old and 35-d-old offspring, no significant differences on the BWG, FI, and the FCR of offspring from 22 to 28 d, 29 to 35 d, and 36 to 42 d (*p >* 0.05; data not shown) were observed among treatments.

The results of serum and liver antioxidant status of offspring are presented in Table 5 and Table 6. The serum T-SOD activity of 42-d-old offspring was higher than that of 21-d-old offspring (*p <* 0.05). However, the liver T-SOD activity and T-AOC of 42-d-old offspring were lower than those of 21-d-old offspring (*p <* 0.05). Maternal dietary VE levels linearly and quadratically increased the serum and liver T-SOD activity of offspring (*p <* 0.05). There were linear and quadratic decreases in liver MDA content of offspring to increasing maternal dietary VE levels (*p <* 0.05). No interaction was found between progeny age and maternal dietary VE on the serum and liver antioxidant status of offspring (*p >* 0.05).

## 4. Discussion

In our study, neither maternal dietary VE levels (100, 200, and 400 mg/kg) nor progeny dietary VE levels (0 and 35 mg/kg) affect the growth performance of offspring hatched from unstored eggs. Consistent with the present study, Hossain et al. [7] and Siegel et al. [28] found that the growth performance of offspring was not influenced by the supplementation of maternal dietary VE. Meanwhile, broiler dietary VE supplementation also showed no significant difference on the growth performance of broilers [29,30,31,32]. The result of the growth performance in trial 1 suggested that the addition of 100 mg/kg VE in broiler breeder diet is sufficient for the normal growth of offspring even without any addition of VE in progeny diet. However, in trial 2 of the present study, we observed that maternal dietary VE supplementation could improve the growth performance of offspring hatched from stored eggs. For the early growth, the VE requirement of chicks mainly dependent on its maternal reserve during embryonic development, which dramatically reduced for the first 2 weeks post-hatch [33]. As a result, we did not observe the performance difference of offspring for the first week post-hatch. However, it appeared to present that the performance difference began at the second week (data not shown). Compared to 100 mg/kg VE, maternal dietary supple-mentation of 200 and 400 mg/kg VE only increased the BWG of offspring from day 15 to 21, and resulted in a corresponding increase in BW of 21- and 42-d-old offspring. Prior to the arising of BW difference, the FCR of offspring from day 8 to 14 was already decreased by the maternal VE levels of 200 and 400 mg/kg (data not shown). It seems that the FCR of offspring was more susceptible to the maternal dietary VE levels. According to Araújo et al. [34], in ovo feeding of VE at 17.5 d of embryonic development increased the relative weight of small intestine and the villus height in duodenum of newly hatched chicks, and decreased the FCR post-hatch (1 to 7, 1 to 14, and 1 to 21 d). In our previous studies, compared to 100 mg/kg VE, maternal dietary supplementation of 200 or 400 mg/kg VE increased the hatchability of prolonged storage eggs [26], but not for that of the unstored eggs (Yang, unpublished data). Correspondingly, in the present study, maternal dietary VE levels of 200 or 400 mg/kg were beneficial to the growth performance of the offspring hatched from prolonged storage eggs, but not for that of the offspring hatched from unstored eggs. It can therefore be stated that dietary VE presented a positive effect when poultry suffered from oxidative stress. Min et al. [35] indicated that dietary supplementation of VE improved the body weight of breeder roosters under oxidative stress induced by dexamethasone. In addition, dietary VE supplementation also improved the performance of broilers under heat stress [36,37].

Commercial poultry production usually faces various stresses, especially oxidative stress. The T-SOD plays a vital role in the conversion of O_2_- into H_2_O_2_. As a stable product of lipid peroxidation, the content of MDA reflects the attacking degree of free radical. The T-AOC reflects the total levels of non-enzymatic antioxidants, such as selenium, ascorbic acid, vitamin E, and carotenoids [1]. In the current study, we interestingly observed that as progeny diet was supplemented with 35 mg/kg VE, maternal dietary VE levels (100, 200, and 400 mg/kg) increased the liver T-AOC of 42-d-old offspring. The reason for this result is not clear, but Zhang [38] also observed that when maternal and progeny diet were both supplied with canthaxanthin, it was beneficial to maximize the antioxidant status of offspring. Except for this results, we found neither maternal dietary VE levels nor progeny dietary VE levels affect the antioxidant status of offspring hatched from unstored eggs, which was consistent with the previous study [29]. However, there was literature that reported that the liver and serum VE concentration were increased by the maternal dietary VE supplementation [39], and the MDA content of broilers was decreased by the maternal or progeny dietary VE levels [39,40]. It is likely that the levels of dietary VE are responsible for the different results of antioxidant status between these studies.

A previous study indicated that the T-SOD activity in the liver of broilers stayed relatively stable after 11 day of age [41], however, the T-SOD activity and T-AOC in the liver of broilers showed a significant downtrend due to aging in the case of long-term egg storage. As egg storage duration was prolonged, the relative gene expression of SOD2 of blastoderms was significantly down-regulated [12], and the antioxidant status of newly hatched chicks was damaged [20]. It seems that oxidative stress happened in the process of egg storage, and it may be one reason for decreased hatchability induced by long-term egg storage. Based on this assumption, we have demonstrated that maternal dietary VE supplementation increased the hatchability of stored eggs and the antioxidant status of embryos and chicks hatched from stored eggs in our previous study [26,27]. In the present study, we further observed that in the case of long-term egg storage, the antioxidant status of offspring was enhanced by the increased maternal dietary VE supplementation. Previous studies indicated that dietary VE supplementation suppressed the MDA content of serum and testicle, and increased the mRNA expression of GSH-Px in oxidative stressed roosters [35,38]. The similar results were also found in the studies of broilers [30,31,39]. In the present study, the same as the variation tendency of growth performance, the treatment difference of antioxidant status also began at the second week, and the effect of maternal dietary VE gradually weakened (data not shown). It is suggested that antioxidant status acts a vital role in maternal dietary VE supplementation to improve the performance of offspring hatched from stored eggs. However, there are 2 limitations (the limited replicates and the unsexed chicks) in the trial 2 of our study. Although 26 chicks per replicate were used in trial 2, the limited replicates (*n* = 5) may lead to overestimation or underestimation the effects of maternal dietary VE levels. As chicks per replicate were not sexed, it is not clear whether gender effect is present in trial 2, and further studies need to be conducted to elucidate this possibility.

As egg storage was commonly conducted in commercial breeder broiler production, the economic value of maternal dietary VE supplementation appeared to be particularly important in relieving the adverse impacts of egg storage. In the case of long-term egg storage, compared with 100 mg/kg VE treatment, adding 400 mg/kg VE in breeder diet could increase the economic value of offspring (approximate 0.5 RMB/bird based on performance difference).

## 5. Conclusions

The present study demonstrated that maternal and progeny dietary VE supplementation did not affect the growth performance of offspring hatched from unstored eggs, while maternal dietary supplementation of 200 or 400 mg/kg could improve the growth performance and antioxidant status of offspring hatched from stored eggs. The suitable VE level for the broiler breeder diet was 400 mg/kg in the case of long-term egg storage.

## Figures and Tables

**Table 1:** The composition and nutrient levels of the basal diet (%, as fed-basis)^1^.

Items	Broiler Breeder	Offspring	
		1 to 21 d	22 to 42 d
Ingredient			
Corn	69.50	50.86	51.07
Soybean meal, 43%	19.00	30.42	22.28
Soybean oil	1.00	2.36	3.90
Wheat flour	-	4.00	6.00
Gluten meal	-	3.00	4.00
Rapeseed meal	-	2.00	3.60
Corn distiller dried grains with solubles	-	3.00	5.00
Calcium Hydrophosphate	1.14	1.76	1.56
Limestone	8.25	1.10	1.12
Sodium chloride	0.30	0.32	0.31
Vitamin and mineral premix^2^	0.50	0.20	0.20
DL-Methionine, 99%	0.11	0.27	0.21
L-Lysine hydrochloride, 98.5%	0.08	0.45	0.51
Threonine, 98.5%	0.02	0.14	0.14
Choline chloride, 50%	0.10	0.12	0.10
Total	100.00	100.00	100.00
Nutritional composition			
Metabolizable energy (kcal/kg)	2780.00	2925.00	3050.00
Crude protein	13.80	21.80	20.00
Available phosphorus	0.30	0.45	0.42
Calcium	3.40	0.95	0.90
Digestible methionine	0.32	0.57	0.52
Digestible lysine	0.66	1.25	1.15
Digestible methionine + cystine	0.53	0.89	0.82
Digestible threonine	0.46	0.81	0.75

^1^Breeder diet: The basal diet without any addition vitamin E was firstly prepared, and then divided into three equal portions. Vitamin E (DL-α-Tocopherol Acetate) was added to each portion at rate of 100, 200, and 400 mg/kg (the analyzed values were 95.6, 211.3, and 419.8 mg/kg). Broiler diet: Basal diet was prepared, divided into two equal portions, and each portion was supplied with 0 and 35 mg/kg (the analyzed values were 10.6 and 40.7 mg/kg) vitamin E (DL-α-Tocopherol Acetate). ^2^ Content per kilogram of diet. Breeders: Vitamin A,12,000 IU; vitamin D3, 4000 IU; vitamin E, 0 mg; vitamin K3, 4.0 mg; thiamin, 3.0 mg; riboflavin, 11.5 mg; pyridoxine, 7.2 mg; vitamin B12, 0.02 mg; folic acid, 10.8 mg; niacin, 47.1 mg; pantothenic acid, 21.6 mg; Biotin, 0.6 mg; iron, 80 mg; copper, 20 mg; manganese, 82.5 mg; zinc, 100 mg; selenium, 0.3 mg; iodine, 1.2 mg. Broilers: vitamin A,10,000 IU; vitamin D3, 4000 IU; vitamin E, 0 mg; vitamin K3, 3 mg; thiamin, 3.5 mg; riboflavin, 10 mg; pyridoxine, 4 mg; vitamin B12, 0.02 mg; folic acid, 2 mg; niacin, 65 mg; pantothenic acid, 15 mg; Biotin, 0.2 mg; iron, 80 mg; copper, 10 mg; manganese, 120 mg; zinc, 100 mg; selenium, 0.3 mg; iodine, 1.0 mg.

**Table 2:** Effects of maternal and progeny dietary vitamin E on the growth performance of offspring hatched from unstored eggs (trial 1) ^1,2^.

Items	Progeny VE 0 mg/kg			Progeny VE 35 mg/kg			Progeny VE		Maternal VE			SEM	*p*-Value		
	Maternal VE 100 mg/kg	Maternal VE 200 mg/kg	Maternal VE 400 mg/kg	Maternal VE 100 mg/kg	Maternal VE 200 mg/kg	Maternal VE 400 mg/kg	0 mg/kg	35 mg/kg	100 mg/kg	200 mg/kg	400 mg/kg		Progeny VE	Maternal VE	Interaction
BW (g/bird)															
1 d	46.63	46.35	46.47	46.54	46.48	46.54	46.48	46.52	46.58	46.42	46.51	0.039	0.654	0.252	0.514
21 d	913.2	899.4	928.3	914.8	916.1	916.0	913.6	915.6	914.0	907.7	922.1	4.208	0.812	0.385	0.385
42 d	2733.8	2701.8	2784.9	2736.2	2728.4	2724.3	2740.2	2729.6	2735.0	2715.1	2754.6	17.708	0.768	0.665	0.590
BWG (g/bird/day)															
1 to 21 d	41.3	40.6	42.0	41.3	41.4	41.4	41.3	41.4	41.3	41.0	41.7	4.210	0.812	0.392	0.383
22 to 42 d	86.7	85.8	88.4	86.8	86.3	86.2	87.0	86.4	86.7	86.1	87.3	15.973	0.705	0.807	0.735
1 to 42 d	128.0	126.4	130.4	128.1	127.7	127.6	128.3	127.8	128.0	127.1	129.0	17.714	0.775	0.659	0.595
FI (g/bird)															
1 to 21 d	1226.7	1213.6	1244.1	1222.3	1223.5	1224.1	1228.1	1223.3	1224.5	1218.6	1234.1	5.635	0.672	0.530	0.562
22 to 42 d	3370.4	3337.2	3398.6	3302.5	3302.9	3301.5	3368.7	3302.3	3336.4	3320.1	3350.1	21.603	0.135	0.852	0.839
1 to 42 d	4583.6	4530.6	4624.8	4506.6	4509.1	4509.1	4579.7	4508.3	4545.1	4519.8	4566.9	24.256	0.151	0.733	0.731
FCR (g:g)															
1 to 21 d	1.417	1.423	1.410	1.408	1.408	1.410	1.417	1.409	1.413	1.416	1.410	0.004	0.318	0.824	0.728
22 to 42 d	1.853	1.853	1.832	1.813	1.823	1.827	1.846	1.821	1.833	1.838	1.829	0.009	0.163	0.912	0.705
1 to 42 d	1.707	1.708	1.690	1.677	1.680	1.685	1.702	1.681	1.692	1.694	1.688	0.006	0.074	0.891	0.611
Mortality (%)															
1 to 21 d	1.67	0.83	1.67	2.50	0.83	0.83	1.39	1.39	2.08	0.83	1.25	0.393	0.999	0.427	0.690
22 to 42 d	0.88	5.26	0.88	3.66	2.68	2.68	2.34	3.01	2.27	3.97	1.78	0.565	0.560	0.266	0.137
1 to 42 d	2.50	5.83	2.50	5.83	3.33	3.33	3.61	4.17	4.17	4.58	2.92	0.669	0.681	0.577	0.220

^1^ Each value represents the mean value of 6 replicates/treatment (*n* = 6). ^2^ BW = body weight, BWG = body weight gain, FI = feed intake, FCR = feed conversion ratio, SEM = pooled standard error of the mean.

**Table 3:** Effects of maternal and progeny dietary vitamin E on the antioxidant status of offspring hatched from unstored eggs (trial 1) ^1,2^.

Items	Progeny VE 0 mg/kg			Progeny VE 35 mg/kg			Progeny VE		Maternal VE			SEM	*p*-Value		
	Maternal VE 100 mg/kg	Maternal VE 200 mg/kg	Maternal VE 400 mg/kg	Maternal VE 100 mg/kg	Maternal VE 200 mg/kg	Maternal VE 400 mg/kg	0 mg/kg	35 mg/kg	100 mg/kg	200 mg/kg	400 mg/kg		Progeny VE	Maternal VE	Interaction
21 d serum															
T-SOD (U/mL)	210.82	211.02	203.79	232.61	240.02	238.59	208.54	237.07	221.71	225.52	221.19	10.215	0.174	0.982	0.968
MDA (nmol/mL)	4.25	3.99	4.53	4.04	4.36	4.48	4.26	4.29	4.14	4.17	4.51	0.108	0.869	0.327	0.544
T-AOC (μmol/mL)	1.12	1.37	1.00	1.24	1.26	1.23	1.17	1.24	1.18	1.32	1.11	0.043	0.377	0.163	0.279
42 d serum															
T-SOD (U/mL)	199.27	205.86	224.87	261.43	257.70	293.77	210.00	270.97	230.35	231.78	259.32	17.909	0.099	0.760	0.981
MDA (nmol/mL)	4.57	4.54	4.46	4.61	4.63	4.28	4.52	4.51	4.59	4.58	4.37	0.119	0.952	0.690	0.876
T-AOC (μmol/mL)	0.86	0.72	0.78	0.78	0.73	0.77	0.79	0.76	0.82	0.72	0.77	0.019	0.436	0.125	0.601
21 d liver															
T-SOD (U/mgprot)	523.48	553.79	554.43	546.49	517.66	524.27	543.90	529.47	534.98	535.72	539.35	10.000	0.476	0.982	0.424
MDA (nmol/mgprot)	1.04	0.80	0.90	0.94	1.06	0.82	0.91	0.94	0.99	0.93	0.86	0.043	0.752	0.485	0.208
T-AOC (μmol/10 mgprot)	1.59	1.66	1.62	1.64	1.57	1.73	1.62	1.65	1.61	1.61	1.68	0.021	0.593	0.370	0.173
42 d liver															
T-SOD (U/mgprot)	398.60	397.80	428.76	402.22	400.52	435.43	408.39	412.72	400.41	399.16	432.09	9.724	0.825	0.307	0.996
MDA (nmol/mgprot)	0.74	0.69	0.79	0.80	0.76	0.71	0.74	0.76	0.77	0.72	0.75	0.024	0.721	0.713	0.375
T-AOC (μmol/10 mgprot)	1.21 ^b,c^	1.21 ^b,c^	1.28 ^b^	1.13 ^c^	1.16 ^b,c^	1.45 ^a^	1.24	1.25	1.17 ^b^	1.19 ^b^	1.37 ^a^	0.017	0.717	<0.001	0.014

^a,b,c^ Different superscripts in the same row indicate significant difference (*p <* 0.05). ^1^ Each value represents the mean value of 6 replicates/treatment (*n* = 6). ^2^ T-SOD = total superoxide dismutase, MDA = malondialdehyde, T-AOC = total antioxidant capacity, SEM = pooled standard error of the mean.

**Table 4:** Effects of maternal dietary vitamin E supplementation on the growth performance of offspring hatched from stored eggs (trial 2) ^1,2^.

Items	Maternal VE (mg/kg)			SEM	*p*-Value		
	100	200	400		Maternal VE	Linear ^3^	Quadratic ^4^
BW (g/bird)							
1 d	49.24	49.19	49.13	0.097	0.915	0.670	0.915
21 d	895.6 ^b^	947.2 ^a^	949.9 ^a^	8.385	0.002	0.013	0.002
42 d	2875.5 ^b^	2930.0 ^a,b^	2958.2 ^a^	13.850	0.031	0.014	0.031
BWG (g/bird/day)							
1 to 21 d	40.4 ^b^	42.8 ^a^	42.9 ^a^	8.327	0.002	0.013	0.002
22 to 42 d	94.4	94.6	95.7	11.207	0.567	0.286	0.567
1 to 42 d	134.7 ^b^	137.4 ^a,b^	138.7 ^a^	13.617	0.027	0.013	0.027
FI (g/bird)							
1 to 21 d	1180.9	1188.1	1170.3	4.657	0.314	0.265	0.314
22 to 42 d	3683.0	3703.0	3763.8	21.024	0.281	0.105	0.281
1 to 42 d	4832.9	4860.8	4896.2	22.336	0.546	0.265	0.546
FCR (g:g)							
1 to 21 d	1.393 ^a^	1.321 ^b^	1.298 ^b^	0.012	<0.001	0.001	<0.001
22 to 42 d	1.859	1.865	1.873	0.006	0.655	0.351	0.655
1 to 42 d	1.708	1.684	1.682	0.005	0.087	0.074	0.087
Mortality (%)							
1 to 21 d	1.54	2.31	3.08	0.628	0.641	0.345	0.641
22 to 42 d	2.65	0.00	2.73	0.588	0.088	0.660	0.088
1 to 42 d	3.85	2.31	5.39	0.839	0.352	0.346	0.352

^a,b^ Different superscripts in the same row indicate significant difference (*p <* 0.05). ^1^ Each value represents the mean value of 5 replicates/treatment (*n* = 5). ^2^ BW = body weight, BWG = body weight gain, FI = feed intake, FCR = feed conversion ratio, SEM = pooled standard error of the mean. ^3^ Linear response to maternal VE. ^4^ Quadratic response to maternal VE.

**Table 5:** Effects of maternal dietary vitamin E supplementation and progeny age on the serum antioxidant status of offspring hatched from stored eggs (trial 2) ^1,2^.

Items	Progeny Age 21 d			Progeny Age 42 d			Progeny Age		Maternal VE			SEM	*p*-Value				
	Maternal VE 100 mg/kg	Maternal VE 200 mg/kg	Maternal VE 400 mg/kg	Maternal VE 100 mg/kg	Maternal VE 200 mg/kg	Maternal VE 400 mg/kg	21 d	42 d	100 mg/kg	200 mg/kg	400 mg/kg		Progeny Age	Maternal VE	Interaction	Linear ^3^	Quadratic ^4^
T-SOD (U/mL)	134.54	130.97	221.27	163.64	196.02	276.22	162.26 ^b^	211.96 ^a^	149.09 ^b^	163.50 ^b^	248.74 ^a^	11.957	0.049	0.005	0.820	0.001	0.005
MDA (nmol/mL)	3.95	3.43	3.56	3.65	3.80	3.29	3.65	3.58	3.80	3.61	3.42	0.114	0.768	0.412	0.404	0.177	0.397
T-AOC (μmol/mL)	1.42	1.30	1.21	1.35	1.19	1.36	1.31	1.30	1.38	1.25	1.28	0.058	0.928	0.613	0.635	0.559	0.589

^a,b^ Different superscripts in the same row indicate significant difference (*p <* 0.05). ^1^ Each value represents the mean value of 5 replicates/treatment (*n* = 5). ^2^ T-SOD = total superoxide dismutase, MDA = malondialdehyde, T-AOC = total antioxidant capacity, SEM = pooled standard error of the mean. ^3^ Linear response to maternal VE. ^4^ Quadratic response to maternal VE.

**Table 6:** Effects of maternal dietary vitamin E supplementation and progeny age on the liver antioxidant status of offspring hatched from stored eggs (trial 2) ^1,2^.

Items	Progeny Age 21 d			Progeny Age 42 d			Progeny Age		Maternal VE			SEM	*p*-Value				
	Maternal VE 100 mg/kg	Maternal VE 200 mg/kg	Maternal VE 400 mg/kg	Maternal VE 100 mg/kg	Maternal VE 200 mg/kg	Maternal VE 400 mg/kg	21 d	42 d	100 mg/kg	200 mg/kg	400 mg/kg		Progeny Age	Maternal VE	Interaction	Linear ^3^	Quadratic ^4^
T-SOD (U/mgprot)	619.43	587.61	637.94	497.42	538.95	603.12	614.99 ^a^	546.50 ^b^	558.43 ^b^	563.28 ^b^	620.53 ^a^	9.743	0.002	0.028	0.167	0.028	0.078
MDA (nmol/mgprot)	0.84	0.72	0.60	0.76	0.82	0.52	0.72	0.70	0.80 ^a^	0.77 ^a^	0.56 ^b^	0.027	0.710	0.002	0.299	<0.001	0.002
T-AOC (μmol/10 mgprot)	1.80	1.71	1.72	1.39	1.30	1.26	1.75 ^a^	1.31 ^b^	1.59	1.51	1.49	0.032	<0.001	0.380	0.945	0.455	0.678

^a,b^ Different superscripts in the same row indicate significant difference (*p <* 0.05). ^1^ Each value represents the mean value of 5 replicates/treatment (*n* = 5). ^2^ T-SOD = total superoxide dismutase, MDA = malondialdehyde, T-AOC = total antioxidant capacity, SEM = pooled standard error of the mean. ^3^ Linear response to maternal VE. ^4^ Quadratic response to maternal VE.

## Data Availability

Data is available upon request from the corresponding authors.
